# It's a good thing that severely hypoxic salmon (*Salmo salar*) have a limited capacity to increase heart rate when warmed

**DOI:** 10.1242/jeb.249594

**Published:** 2025-03-06

**Authors:** Anthony K. Gamperl, Julie J. H. Nati, Kathy A. Clow, Rebeccah M. Sandrelli, Lucie Gerber, Emma S. Porter, Ellen C. Peroni

**Affiliations:** Department of Ocean Sciences, Memorial University of Newfoundland and Labrador, St. John's, NL A1C 5S7, Canada

**Keywords:** Climate change, Fish, Heart, Cardiorespiratory function, Thermal tolerance, Hypoxia, Oxygen extraction

## Abstract

With climate change, fish are facing rising temperatures, an increase in the frequency and severity of heat waves and hypoxia, sometimes concurrently. However, only limited studies have examined the combined effects of increases in temperature and hypoxia on fish physiology and survival. We measured the cardiorespiratory physiology of 12°C-acclimated Atlantic salmon when exposed acutely to normoxia [100% air saturation (sat.)] versus 75 and 50% air sat., and then warmed to their critical thermal maximum (CT_max_) at 2°C h^−1^. Fish exposed to 50% air sat. became bradycardic, were unable to increase heart rate (*f*_H_) when warmed, and had lower values for metabolic scope and CT_max_ (21.3 vs 26.1°C in normoxic fish). The effects of 75% air sat. on cardiorespiratory parameters and CT_max_ were intermediate. We then used atropine (1.2 mg kg^−1^) and 8-cyclopentyltheophylline (CPT; 50 nmol kg^−1^) to investigate what role(s) cholinergic tone on the heart and cardiac adenosinergic effects, respectively, play in preventing severely hypoxic salmon (40% air sat.) from increasing *f*_H_ when warmed. CPT had no/limited effects on salmon cardiorespiratory parameters and thermal tolerance. However, atropine increased *f*_H_ in hypoxic fish and allowed it to rise with temperature, and this resulted in salmon that were much less tolerant to warming. Collectively, these results: (1) show that fish in severely hypoxic environments will be very susceptible to climate change-associated heat waves; and (2) suggest that cholinergic tone on the heart is not removed when severely hypoxic fish are exposed to rising temperatures to protect the heart's pumping capacity.

## Teaser

**Summary:** Salmon that initiate hypoxic bradycardia cannot increase heart rate when warmed. This response is mediated by cholinergic tone and protects heart function.

## INTRODUCTION

As a result of climate change, marine species are facing a number of changes in water physiochemical properties [i.e. pH, salinity, temperature and oxygen levels; Garcia-Soto et al., 2021; IPCC, 2022). Two of the most challenging of these are increases in temperature and hypoxia (low water oxygen levels). This is because they are both oxygen-limiting conditions and oxygen is essential to the survival of the vast majority of animals and a key environmental factor that influences the physiology, distribution and survival of water-breathing organisms (Deutsch et al., 2015, 2024; Howard et al., 2020; Sampaio et al., 2021; Stramma et al., 2012). Given concerns about the effects of increasing ocean temperatures and heat waves on fish populations (Frölicher and Laufkötter, 2018; Lefevre et al., 2021; Little et al., 2020; Seebacher et al., 2023; Sinclair et al., 2016), the scientific community has been determining the upper thermal tolerance(s) of numerous species (e.g. see Desforges et al., 2023). They have also been examining how this parameter relates to oxygen consumption (*Ṁ*_O_2__) and cardiac function as heart rate (*f*_H_) and cardiac output (*Q̇*; the amount of blood pumped per minute) increase with temperature, and cardiac collapse appears to be a key factor in determining the upper temperature limit of fishes (Eliason and Anttila, 2017; Farrell et al., 2009; Haverinen and Vornanen, 2020; Kuzmin et al., 2022; Vornanen, 2020; Wang and Overgaard, 2007). In addition, several studies have examined the effects of acute and/or chronic hypoxia on various aspects of fish cardiorespiratory function (Carenvale et al., 2021; Gerber et al., 2019; Leeuwis et al., 2019; Morgenroth et al., 2021; Motyka et al., 2017; Petersen and Gamperl, 2010a,b).

**List of symbols and abbreviations**

AS: aerobic scope
AV: atrioventricular
cAMP: cyclic adenosine monophosphate
*C*a_O_2__: arterial blood oxygen content
CPT: 8-cyclopentyltheophylline
CT_max_: critical thermal maximum
CTT: capillary transit time
CTTH: capillary transit time heterogeneity
*C*v_O_2__: venous blood oxygen content
CVP: central venous pressure
*f*_H_: heart rate
IU: international units
MMR: maximum metabolic rate
*Ṁ*_O_2__: oxygen uptake
*Ṁ*_O_2__/*Q̇*: tissue oxygen extraction
NANC: non-adrenergic non-cholinergic
OEF: fraction of oxygen extracted
OMZ: oxygen minimum zone
*P*_crit_: critical oxygen tension
PCT_max_: partial pressure of oxygen at CT_max_
*P*_O_2__: partial pressure of oxygen
*P*w_O_2__: partial pressure of oxygen in water
*Q̇*: cardiac output
RVM: relative ventricular mass
*V*_s_: stroke volume

However, these two environmental challenges can co-occur, and this represents a particularly difficult situation for aquatic organisms, including fishes. For example, warm temperatures increase an organism's metabolic demand and simultaneously increase the severity of environmental hypoxia (i.e. warm water holds less oxygen). To date, there are only a few studies that have directly examined how the combined stressors of hypoxia and high temperature constrain the capacity of the cardiorespiratory system to meet a fish's metabolic need *in vivo*, and how this is related to their upper thermal tolerance (Burleson and Silva, 2011; Motyka et al., 2017; Leeuwis et al., 2021). Interestingly, Leeuwis et al. (2021): (1) showed that hypoxia-induced bradycardia prevented the sablefish (*Anaplopoma fimbria*) from increasing *f*_H_ when exposed to increasing temperatures; and (2) suggested that this physiological constraint severely limits a fish's cardiac response to the latter stressor, and ultimately blood oxygen transport capacity and thermal tolerance. Such information is critical to understanding how fish will respond to these two challenges in nature as cardiac (*f*_H_) failure is thought to be functionally, and more ecologically, relevant than the temperature at which the fish loses equilibrium [i.e. reaches its critical thermal maximum (CT_max_)] (Sandrelli and Gamperl, 2023; Sidhu et al., 2014). However, the sablefish is a species that lives in oxygen minimum zones (OMZs; areas with O_2_ levels of less than 2 mg l^−1^) as adults (Doya et al., 2017; Mason et al., 1983) and is extremely hypoxia tolerant [critical oxygen tension (*P*_crit_) of 15.8% air sat. at 10°C; Leeuwis et al., 2019], and thus, it is not known how the results of Leeuwis et al. (2021) apply to other (less hypoxia tolerant) fishes.

Thus, we used the Atlantic salmon as a model hypoxia-intolerant species (Barnes et al., 2011; Sandrelli and Gamperl, 2023) to examine aspects of fish cardiorespiratory physiology when exposed to acute hypoxia followed by an increase in temperature to the fish's CT_max_. Specifically, we asked four questions. (1) Does mild hypoxia (75% air sat.; i.e. prior to reaching an oxygen level where this species goes bradycardic) affect the salmon's *f*_H_–temperature relationship and CT_max_? (2) Once hypoxia is severe enough that this species goes bradycardic (i.e. at ∼50% air sat.), does this prevent them from increasing *f*_H_ when warmed? Given that the answer to question 2 was yes: (3) what roles do cholinergic innervation (tone) and adenosinergic modulation of cardiac function play in preventing the temperature-dependent increase in *f*_H_ in Atlantic salmon? And (4) does this inability to increase *f*_H_ limit this species' ability to meet temperature-induced metabolic demands and result in a decrease in thermal tolerance? Question 3 is important given that both cholinergic and adenosinergic mechanisms can influence *f*_H_ in fishes, and are known to induce bradycardia in this taxon (Aho and Vornanen, 2002; Gamperl and Driedzic, 2009; Sundin and Nilsson, 1996; Wood and Shelton, 1980).

## MATERIALS AND METHODS

### Animals

This study was approved by the Animal Care Committee of Memorial University (protocol ^#^19-01-KG), and salmon husbandry and experimental procedures were performed in accordance with the Canadian Council on Animal Care Guidelines on the ‘Care and Use of Fish in Research, Teaching and Testing’ (2005).

Atlantic salmon (*Salmo salar* Linnaeus 1758) of Saint John River origin were obtained from commercial aquaculture companies as smolts and initially maintained at the Dr Joe Brown Aquatic Research Building (JBARB, Memorial University of Newfoundland) in 3000 liter tanks at 8–10°C. These fish were then transferred to 2200 liter circular tanks receiving 12°C (experiment 1) or 10°C (experiment 2) (±1°C) aerated seawater and a 14 h light:10 h dark photoperiod at the Laboratory for Atlantic Salmon and Climate Change Research (LASCCR, Ocean Sciences Centre, St. John's, Newfoundland, Canada). Fish were fed a commercial marine fish diet (EWOS Dynamic S) at ∼0.7% body mass day^−1^ while being held in the LASCCR, except for the day before the fish were used for experiments. After several months of acclimation to these tanks, and ∼24 h after they had been fed, 1 or 2 fish of similar size were netted from the tanks every other day for the experiments described below.

### Surgery

Salmon (experiment 1, 1061±48 g; experiment 2, 1499±39 g; mean±s.e.m.) were initially anaesthetized with tricaine methansulfonate (TMS, 0.2 g l^−1^; Syndel, Nanaimo, BC, Canada) and an equal amount of bicarbonate in oxygenated seawater until ventilatory movements ceased. The fish were then quickly weighed and measured for length, and placed on their right side on a surgical table where their gills were irrigated with cooled (8–10°C) and oxygenated seawater containing a maintenance dose of TMS (0.1 g l^−1^). The opercular cavity was opened, and a small hole was made in the skin above the position of the ventral aorta. This hole was expanded using blunt dissection and an appropriate sized (1.6–2 mm depending on size of fish) Doppler^®^ flow probe (Model ES Cuff-type Transducer, 20 MHz, Iowa Doppler Products, Iowa City, IA, USA) was placed on the aorta. Once the probe was in place, the probe cuff was tied shut and the leads were connected to a directional pulsed Doppler^®^ flow meter (Model 545C-4; Bioengineering, University of Iowa, Ames, IA, USA) that was interfaced with a MP100A-CE data acquisition system and a laptop running AcqKnowledge^®^ software (BIOPAC Systems Inc., Santa Barbara, CA, USA) to check signal quality. Finally, the leads were sutured to the fish at three different locations, just inside the opercular cavity, below the pectoral fin and beside the dorsal fin.

In addition, salmon in experiment 2 were fitted with a PE50 (Becton Dickinson, Fair Lakes, NJ, USA) dorsal aortic cannula (Eliason et al., 2022; Smith and Bell, 1964) to allow for intra-arterial injections of saline and/or pharmacological agents. This cannula was secured to the fish just anterior to the dorsal fin. This cannula was filled with 0.9% NaCl (saline) containing 100 IU ml^−1^ of sodium heparin and flushed with saline containing 10 IU ml^−1^ of heparin periodically, to keep the cannula patent. For an illustration of the surgical procedures used in these experiments, see Lee et al. (2003). Fig. S1 shows the inside of the salmon's opercular cavity after implantation of the blood flow probe was complete.

Following surgery, the fish were placed into a respirometry chamber (see below) submerged in a water table at 12°C (experiment 1) or 10°C (experiment 2) and allowed to recover overnight. The water table was surrounded by black screening so that the fish could not see the experimenters and noise level was kept to a minimum.

### Respirometry

A large reservoir (∼300 liters) with seawater at 12°C or 10°C and 95–100% air sat. supplied a water table that contained two Plexiglass 19.8 liter respirometers (20.3 cm in diameter×61.0 cm long). The O_2_ level in the water was regulated by a control system (OXY-REG, Loligo Systems; Tjele, Denmark) that monitored the O_2_ level with a galvanic O_2_ probe (MINI-DO) and made adjustments to the water's O_2_ level by bubbling O_2_ or N_2_ gas into the reservoir. The water temperature in the reservoir was controlled primarily by a custom-made heater/chiller (Technical Services, Memorial University), but supplemented by two additional circulating water baths (model 1013S; Fisher Scientific, Pittsburgh, PA, USA). Two submersible pumps (model 1048; EHEIM GmbH & Co., Deizisau Germany) in the water table were connected to each respirometer and controlled by AutoResp^®^ software (version 2.2.2; LoligoSystems) allowing the respirometers to be switched between ‘flushing’ (open) and ‘recirculating’ (closed) modes.

Measurements of oxygen consumption (*Ṁ*_O_2_;_ in mg O_2_ kg h^−1^) were made considering the recommendations for aquatic respirometry as detailed in Killen et al. (2021), Rodgers et al. (2016) and Svendsen et al. (2016). Oxygen in the respirometers was measured using dipping probes that were pre-calibrated in the lab at 0% and 100% air saturation, a fibre-optic OXY-4 mini oxygen meter (PreSens, Regensburg, Germany) interfaced with DAQ-4 and TEMP-4 modules (LoligoSystems) and a computer running AutoResp^®^ software (LoligoSystems). Oxygen consumption was measured in the respirometers at various oxygen levels/temperatures by stopping the flow of seawater into the respirometers (i.e. the flush pump was switched off) for 5–7 min (depending on temperature), which included a 1–2 min ‘wait’ period before the AutoResp^®^ software would start collecting data. The software automatically calculated the fish's *Ṁ*_O_2_ _using the slope of the relationship between time and water O_2_ content and taking into account the volume that the fish occupied in the respirometer. Once the *R*^2^ was greater than 0.95 or if the O_2_ decreased by ∼5% of the initial value, the respirometers were manually put into the ‘flush’ mode to restore the oxygen level in the respirometer. Both ƒ_H_ and cardiac output (*Q̇*; volts) were recorded continuously.

Note that water O_2_ levels were measured several times in the respirometers with no fish present and oxygen consumption was negligible (<0.5% of the resting *Ṁ*_O_2__ of the fish), and thus no corrections were made for background respiration.

### Experiment 1

#### Preliminary hypoxic challenge experiment

Before running the first thermal challenge experiment (experiment 1), we needed to determine the oxygen level at which these salmon initiate bradycardia at 12°C. Thus, a preliminary acute hypoxic challenge was performed on 8 adult salmon (890±70.1 g). Approximately 20 h after surgery, the Doppler^®^ flow probe leads from two fish were connected to the Doppler^®^ flow meter. The salmon were then allowed to rest for at least 1 h, and once cardiac parameters were stable, *f*_H_ and *Ṁ*_O_2__ were recorded, and the chamber was ‘closed’. Cardiac function was measured continuously and *Ṁ*_O_2__ was recorded at every 5% decrease in water O_2_ content until the level of oxygen in the respirometer reached 40% of air saturation. At this point, the respirometers were returned to the ‘flushing’ mode (i.e. to normoxic conditions). This experiment took approximately 1.5–2 h to complete.

#### Thermal challenge experiment

In the preliminary experiment, no fish initiated bradycardia prior to reaching 70% air saturation and mean ƒ_H_ had decreased significantly when water O_2_ levels reached 55% air sat. (see Fig. S2). Thus, the levels of hypoxia chosen for this experiment were 75% and 50% air sat. [a level of hypoxia where all fish were bradycardic and that is approximately 10% above the Atlantic salmon's *P*_crit_ at this temperature (35–40% air sat. at 12°C; Remen et al., 2013, 2016)]. As with the preliminary study, two salmon were instrumented and placed in the respirometers, and the next morning the Doppler^®^ flow leads were connected to the flow meter and the fish were allowed to settle for at least 1 h at 12°C. Once ƒ_H_ was stable, the respirometers were ‘closed’ and the AutoResp^®^ software measured the fish's oxygen consumption/metabolic rate.

Thereafter, the fish were randomly assigned to one of the three treatments (100, 75 or 50% air sat.; *N*=9 per treatment) and the seawater's oxygen level lowered (if required) over a 1 h period by decreasing water O_2_ levels in the reservoir and water table. After an additional hour at their assigned oxygen level, the *Ṁ*_O_2__ and cardiac function of the fish were recorded again, and then temperature was set to increase at 2°C h^−1^. *Ṁ*_O_2__, *f*_H_ and *Q̇* were recorded at 14°C, 16°C and at every 1°C thereafter until the fish lost equilibrium (i.e. reached its CT_max_). Since two fish were run at the same time, the fish that reached its upper temperature tolerance first was euthanized immediately by injecting 0.3 g l^−1^ TMS into its respirometer while in the recirculation/‘closed’ mode. This ensured that the fish that reached its CT_max_ first was not in distress while the experiment on the fish with the higher thermal tolerance was completed. The specific protocol used in this experiment is shown in Fig. S3.

### Experiment 2

Based on experiment 1, it was clear that the Atlantic salmon were not able to increase their ƒ_H_ at 50% air sat. (after they went bradycardic) when warmed to their CT_max_. Thus, we used a similar protocol to examine the physiological (mechanistic) basis of this phenomenon (see Fig. S4). The basic protocol used for the first (control; *N*=7) group for this experiment was similar to that for the severely hypoxic group in experiment 1 except that: (1) the water O_2_ level chosen for hypoxia was 40% air sat. as the fish were acclimated to a colder temperature (10°C) and fish hypoxia tolerance is temperature dependent (Remen et al., 2013); (2) the salmon were injected with 0.5 ml of saline after reaching this level of hypoxia and at 14 and 18°C (see below for reasoning); and (3) temperature was only increased to 20°C. However, in this experiment, there were two additional groups. In group 2 (*N*=7), atropine sulfate (cholinergic nerve blocker; 1.2 mg kg^−1^) was injected into the fish after it had been at 40% air sat. for 1 h, and these fish were injected with saline at 14 and 18°C. In group 3 (*N*=8), CPT (8-cyclopentyltheophylline; A_1_-receptor antagonist, 50 nmol kg^−1^) was injected at 1 h after the fish reach 40% air sat. and when the fish reached 14°C and 18°C. CPT was injected three times as it has been previously shown that the effects of this A_1_-receptor antagonist are short-lived in fishes, that cardiovascular variables stabilise quickly after injection (Sundin et al., 1999), and that the effects of adenosine on the trout (*Oncorhynchus mykiss*) heart are dependent on temperature (Aho and Vornanen, 2002). These two pharmacological agents were purchased from Sigma-Aldrich Chemical (Oakville, ON, Canada), and measurements were taken at 15 min post-injection.

### Data and statistical analyses

Heart rate (*f*_H_; beats min^−1^) was determined by measuring the time required for 20 peaks in the blood flow trace at two points at each temperature. Because *Q̇* was dependent on the particular probe used and the position/placement of the probe, values for *Q̇* and *V*_s_ are presented as a percentage of their values at the initial measurement point (i.e. 10°C or 12°C; with all fish at normoxia). Relative stoke volume (*V*_s_) was calculated as *Q̇*/*f*_H_, and oxygen extraction was calculated (in %) as *Ṁ*_O_2__/*Q̇*. The absolute scope for all parameters in experiment 1 was calculated by subtracting the value recorded for each fish at 12°C at 100, 75 or 50% air sat. from its maximum value during the temperature challenge.

To determine the mean oxygen level at which *f*_H_ decreased significantly (i.e. the oxygen level at which the fish became bradycardic) in the preliminary experiment, a repeated measures one-way ANOVA followed by Dunnett's tests was performed. Differences between initial (at 100% air sat.) cardiorespiratory parameters and those measured at the fish's experimental O_2_ level in experiment 1 were identified using a paired *t*-test. One-way ANOVAs followed by Tukey's multiple comparisons tests were also performed to compare various physiological parameters (*f*_H_, *Q̇*, *V*_s_, *Ṁ*_O_2__, *Ṁ*_O_2__/*Q̇,* CT_max_) between groups (Table 1). In experiment 2, significant differences between initial values (at 100% air sat.) and those measured at 40% air sat. were also identified using paired *t*-tests. In addition, two-way repeated measures ANOVAs, with temperature and group as fixed factors, were used to compare parameters between groups, and between temperatures within a group, up to 18°C (Table 2). Linear regressions were performed between various cardiorespiratory parameters and CT_max_ to identify significant relationships overall, and within specific groups in experiment 1. All statistical analyses were performed using Prism software (GraphPad; Irvine, CA, USA), and with *P*<0.05 as the level of significance. All values reported in the text, figures and tables are means±1 standard error (s.e.m.).

**Table 1.** **Cardiorespiratory parameters in Atlantic salmon in normoxia (100% air saturation) at 12°C (*N*=7), when two groups were exposed to hypoxia (75 and 50% air saturation; *N*= 8 and *N*=10, respectively), and then when all three groups were warmed to their critical thermal maximum (CT_max_) at 2°C** **h^−1^**

		Normoxia	Hypoxia	Change	Maximum	Scope
100%	*f*_H_	77.6±3.5^a^	78.0±2.6^a^	0.4±1.7^a^	126.3±6.1^a^	48.2±5.8^a^
	*Q̇*	100.0±0.0	98.6±1.8^a^	−1.4±1.8^a^	149.7±8.7^a^	51.0±8.5^a^
	*V*_s_	100.0±0.0	97.34±2.4^a^	−2.7±2.4^a^	112.7±6.2^a^	15.4±6.1^a^
	*Ṁ*_O_2__	148.7±21.9^a^	130.4±16.7^a,‡^	−18.3±5.9^a^	320.1±12.5^a^	189.7±22.0^a^
	*Ṁ*_O_2__/*Q̇*	100.0±0.0	89.9±3.1^a^	−10.1±3.1^a^	182.0±30.2^a^	92.1±27.3^a^
75%	*f*_H_	80.8±1.4^a^	78.0±1.4^a^	−2.9±1.5^a^	113.6±4.4^a^	35.6±3.9^a^
	*Q̇*	100.0±0.0	113.6±3.5^a^	13.6±3.5^a^	157.0±11.1^a^	43.4±11.8^a,b^
	*V*_s_	100.0±0.0	118.0±4.2^a^	18.0±4.2^a^	131.4±5.2^a^	13.4±4.9^a^
	*Ṁ*_O_2__	98.4±7.4^b^	107.4±13.3^a^	9.1±13.1^a^	210.6±11.0^b^	103.2±14.6^b^
	*Ṁ*_O_2__/*Q̇*	100.0±0.0	99.1±10.5^a^	−0.9±10.5^a^	180.8±19.2^a^	81.6±20.8^a^
50%	*f*_H_	80.0±1.1^a^	57.0±3.5^b*^	−23.0±3.2^b^	77.9±3.9^b^	20.9±2.8^b^
	*Q̇*	100.0±0.0	108.4±5.9^a^	8.4±5.9^a^	141.8±9.8^a^	33.4±7.0^b^
	*V*_s_	100.0±0.0	157.0±12.2^b^	57.0±12.2^b^	189.8±12.0^b^	32.7±10.0^a^
	*Ṁ*_O_2__	103.4±8.1^a,b^	117.8±12.2^a^	14.4±10.3^a^	193.4±7.8^b^	75.6±18.6^b^
	*Ṁ*_O_2__/*Q̇*	100.0±0.0	112.8±13.4^a^	12.8±13.4^a^	166.9±15.0^a^	54.1±15.2^a^

Heart rate (*f*_H_) is in beats min^−1^ and oxygen consumption (*Ṁ*_O_2__) is in mg O_2_ kg^−1^ h^−1^, whereas cardiac output (*Q̇*) and stroke volume (*V*_s_) are in percentages (100%=the initial value for each fish under normoxia). *Ṁ*_O_2__/*Q̇* has units of mg O_2_ kg^−1^ h^−1^%^−1^. ‘Change’ is the difference between normoxic (initial) values and those for hypoxic fish at 12°C. Note that normoxic fish (i.e. 100%) were not exposed to hypoxia (i.e. this group was the control group). ‘Maximum’ is the highest value recorded for each fish, whereas ‘scope’ is the change in a parameter during the CT_max_ portion of the experiment. Dissimilar lowercase letters indicate a significant (*P*<0.05) difference between groups (one-way ANOVAs followed by Tukey's HSD tests); *P<0.05 and ^‡^*P*<0.10 between fish exposed to normoxia and hypoxia at 12°C (paired t-tests). Note that all ‘hypoxia’ and ‘maximum’ values were significantly different (*P*<0.05). Data are means±s.e.m.

**Table 2.** **Cardiorespiratory parameters in Atlantic salmon in normoxia (100% air saturation) at 10°C, when exposed to 40% air saturation, and warmed to 20°C (at 2°C** **h^−1^) after injection with saline (Control group, *N*=7), atropine (1.2 mg** **kg^−1;^ *N*=7) or 8-cyclopentyltheophylline (CPT; 50 nmol** **kg^−1^, *N*=8)**

		Normoxia	Hypoxia				
		10°C	10°C	10°C 15 min post-injection	14°C	16°C	18°C
Saline	*f*_H_	76.3±2.1^a^	47.7±7.1^a,A*^	34.4±3.8^a,A^	39.7±4.1^a,A^	41.6±3.1^a,A^	48.7±4.8^a^ (6)
	*Q̇*	100.0±0.0	96.4±7.8^a,A^	90.8±7.7^a,A^	94.0±9.4^a,A^	97.9±10.4^a,A^	93.5±15.3 (6)
	*V*_s_	100.0±0.0	168.8±20.2^a,A^	208.0±17.5^a,A^	186.2±17.2^a,A^	184.6±22.4^a,A^	154.7±28.2 (6)
	*Ṁ*_O_2__	82.9±6.7^a^	82.4±4.7^a,A^	76.3±2.5^a,A^	72.1±2.1^a,A^	71.8±3.2^a,b,A^	72.3±4.6^a^ (6)
	*Ṁ*_O_2__/*Q̇*	100.0±0.0	110.5±12.5^a,b,A^	108.4±10.3^a,A^	100.3±11.4^a,b,A^	94.0±6.6^a,A^	106.0±12.2 (6)
Atropine	*f*_H_	79.4±1.4^a^	42.3±4.7^a,A,*^	68.6±1.8^b,B^	76.0±3.5^b,B,C^	78.9±3.6^b,C^	97.8±6.7^b^ (4)
	*Q̇*	100.0±0.0	98.8±3.3^a,A^	121.5±4.4^b,A^	108.3±11.5^a,A^	89.7±9.7^a,B^	87.6±26.0 (3)
	*V*_s_	100.0±0.0	196.1±18.8^a,A^	141.3±7.2^b,B^	111.1±7.5^b,B^	88.9±7.2^b,C^	74.8±22.5 (3)
	*Ṁ*_O_2__	99.7±7.9^a^	81.3±5.3^a,A,*^	77.2±3.1^a,A^	70.8±4.2^a,**A**^	66.6±5.0^a,**A**^	69.9±0.7^a^ (4)
	*Ṁ*_O_2__/*Q̇*	100.0±0.0	84.9±7.3^a,A^	67.2±7.4^b,B^	73.2±11.5^a,A^	82.4±11.3^a,A^	91.6±21.8 (3)
CPT	*f*_H_	75.2±2.2^a^	43.5±4.6^a,A,*^	39.7±3.6^a,A^	39.0±2.1^a,A^	41.9±2.2^a,A^	44.3±2.2^a^ (8)
	*Q̇*	100.0±0.0	86.9±6.6^a,A^	86.1±7.2^a,A^	85.5±7.8^a,A^	81.2±6.8^a,A^	83.6±9.9 (8)
	*V*_s_	100.0±0.0	161.2±19.7^a,A^	169.2±16.3^a,b,A^	165.3±13.1^a,A^	146.8±11.4^a,A^	140.0±13.2 (8)
	*Ṁ*_O_2__	76.2±6.1^a^	91.2±3.4^a,A,*^	84.8±5.0^a,A^	78.9±3.3^a,A^	83.4±4.1^b,A^	85.1±6.7^a^ (8)
	*Ṁ*_O_2__/*Q̇*	100.0±0.0	145.1±9.5^b,A^	135.6±8.6^a,A^	130.9±13.7^b,A^	145.0±14.8^b,A^	145.8±13.1 (8)

Heart rate (*f*_H_) is in beats min^−1^ and oxygen consumption (*Ṁ*_O_2__) is in mg O_2_ kg^−1^ h^−1^, whereas cardiac output (*Q̇*) and stroke volume (*V*_s_) are in percentages (100%=the initial value for each fish under normoxia). *Ṁ*_O_2__/*Q̇* has units of mg O_2_ kg^−1^ h^−1^%^−1^. *P<0.05 between 10°C non-injected fish at 100% air saturation (normoxia) versus at 40% air saturation (hypoxia) determined using paired t-tests. Dissimilar lowercase letters indicate a significant difference between groups at a particular time point in the experiment (one-way ANOVAs), whereas dissimilar capital letters indicate differences within a treatment before and after drug administration (one-way repeated measures ANOVAs). This analysis was only performed up to 16°C given the limited number of fish in the atropine-injected group after this temperature. At 18°C, between group comparisons are only made when *N*=4 in the atropine group. Data are means±s.e.m. In the final column, numbers in parentheses indicate fish remaining at this temperature.

## RESULTS

### Experiment 1

**Fig. 1.**
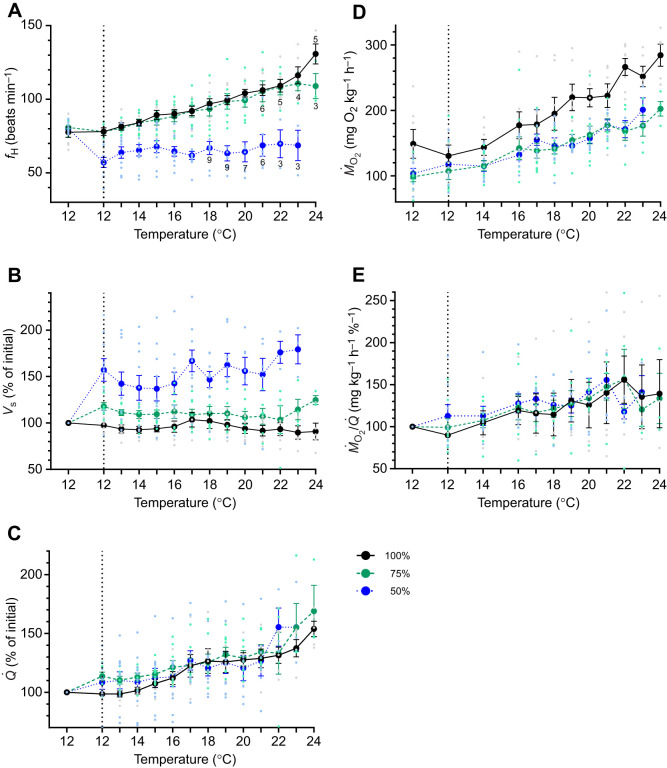
**Effect of hypoxia and increased temperature on cardiorespiratory parameters in Atlantic salmon.** (A) heart rate (*f*_H_), (B) stroke volume (*V*_s_), (C) cardiac output (*Q̇*), (D) oxygen consumption (*Ṁ*_O_2__) and (E) tissue oxygen extraction (*Ṁ*_O_2__/*Q̇*) in normoxia (100% air saturation, *N*=7) at 12°C, when two groups were exposed to hypoxia (75 and 50% air saturation, *N*=8 and 10, respectively; vertical dotted line) and then when all three groups were warmed to their critical thermal maximum (CT_max_) at 2°C h^−1^. In A, numbers indicate how many fish were remaining if less than started the trial. Note that data with *N*≤3 are not plotted. Values are means±s.e.m. Light symbols show the data points for individual fish.

In the control group, and fish exposed to 75% air sat., none of the cardiorespiratory parameters changed significantly (i.e. *P*>0.05) prior to warming. However, in fish exposed to 50% sat., *f*_H_ decreased by 29% (from 80 to 57 beats min^−1^) and this was compensated for by an increase in *V*_s_ such that *Q̇* and *Ṁ*_O_2__ were unchanged (Fig. 1, Table 1). In the control (normoxic) group, *f*_H_, *Q̇* and *Ṁ*_O_2__ increased gradually as temperature was raised (Fig. 1), and maximum values for these parameters were ∼48 beats min^−1^ (60%), 50% and 90% higher than those at 12°C, respectively (Table 1). Stroke volume did not change as temperature was increased, and thus, it was the combination of the increase in *f*_H_ and oxygen extraction (by ∼1.9-fold) that allowed for the magnitude of increase in *Ṁ*_O_2__ in the control group (Fig. 1, Table 1). The pattern of temperature-dependent changes in cardiac function and *Ṁ*_O_2__/*Q̇* were similar in fish exposed to 75% air sat. However, the maximum temperature-induced *Ṁ*_O_2__ was 33% less than in control fish (Fig. 1D, Table 1).

At 12°C, salmon held at 50% air sat. had much lower and higher values for *f*_H_ and *V*_s_ (by ∼27 and 61%), respectively, as compared to control fish (Table 1, Fig. 1B,C)_._ These hypoxic fish also had a much lower scope for *f*_H_ when warmed (by 68%), and this resulted in scopes for *Q̇* and *Ṁ*_O_2__ that were significantly lower than in normoxic fish (Table 1). In contrast, values for maximum *Ṁ*_O_2__/*Q̇* and scope for *Ṁ*_O_2__/*Q̇* were not significantly different between fish warmed to their CT_max_ under normoxia versus 50% air sat. (Table 1, Fig. 1E). The CT_max_ of fish warmed at 50% air sat. (21.3±0.7°C) was ∼4.8°C lower than that measured in normoxic fish (26.1±0.7°C), whereas that for fish tested at 75% air sat. was intermediate (23.6±0.9°C) and not significantly different from the other two groups (also see Fig. S5).

**Fig. 2.**
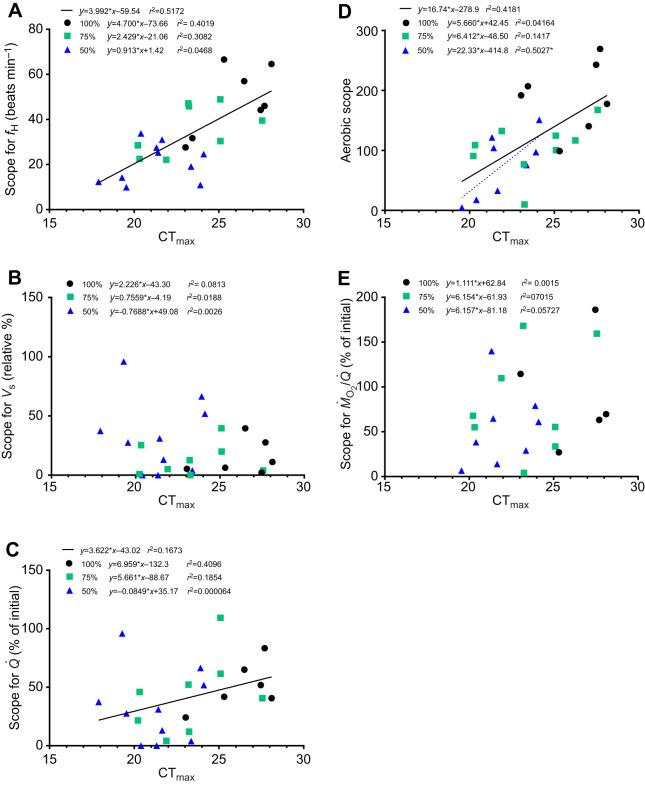
**Relationship between the CT_max_ of fish held under normoxia and hypoxia, and the scope for various cardiorespiratory variables.** Relationships (see dotted lines) between the CT_max_ of fish held under normoxia (*N*=7), and at 75 (*N*=8) and 50% hypoxia (*N*=10). (A) Heart rate (*f*_H_), (B) stroke volume (*V*_s_), (C) cardiac output (*Q̇*), (D) oxygen consumption (*Ṁ*_O_2__) and (E) tissue oxygen extraction (*Ṁ*_O_2__/*Q̇*). The relationships for each parameter when the groups are combined are also shown (see solid lines). Equations for all the linear regressions are shown. However, lines are only shown for those relationships that were significant (*P*<0.05).

As part of our analysis, we examined how the scope for cardiorespiratory parameters [*f*_H_, *V*_s_, *Q̇*, *Ṁ*_O_2_ _(aerobic scope, AS) and *Ṁ*_O_2__/*Q̇*] was related to the salmon's thermal tolerance (CT_max_) both within and across groups (Fig. 2). The only relationship that was significant for a particular group was between AS and CT_max_ for the fish exposed to 50% air sat. (Fig. 2C). When the data for all groups were combined, this relationship was also significant. Furthermore, scope for both *f*_H_ and *Q̇* were positively related to CT_max_ (Fig. 2A,C).

### Experiment 2

**Fig. 3.**
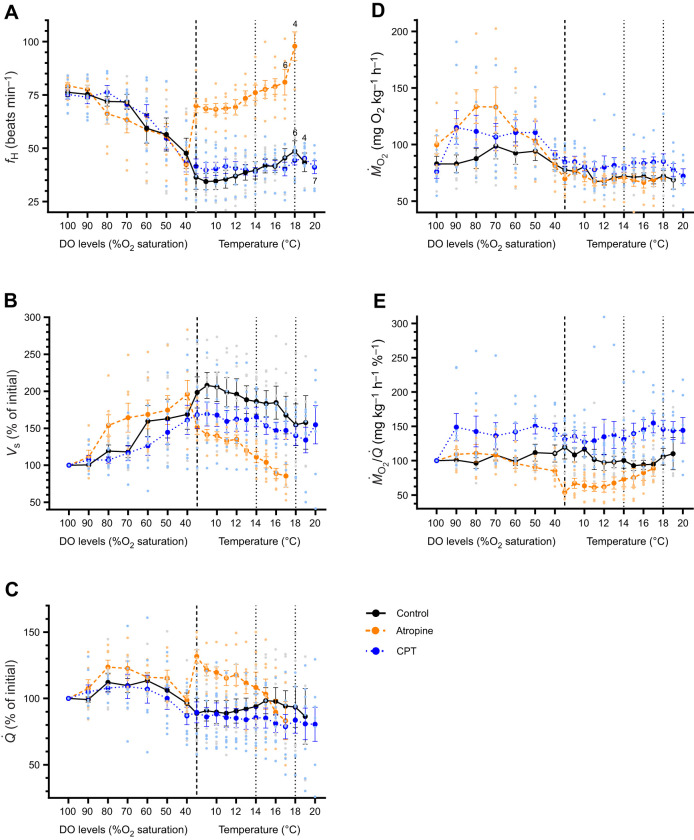
**Effect of atropine and 8-cyclopentyltheophylline under different oxygen saturation and temperature regimes on cardiorespiratory parameters.** Cardiorespiratory parameters in Atlantic salmon in normoxia (100% air saturation, *N*=7) at 10°C, when the water oxygen level was slowly (over 1 h) lowered to 40% saturation, and when warmed to 20°C (at 2°C h^−1^) after being injected with saline (control group; *N*=7) or atropine (1.2 mg kg^−1;^ *N*=7) prior to warming, or 8-cyclopentyltheophylline (CPT; 50 nmol kg^−1^, *N*=8) prior to warming and at 14 and 18°C. (A) heart rate (*f*_H_), (B) stroke volume (*V*_s_), (C) cardiac output (*Q̇*), (D) oxygen consumption (*Ṁ*_O_2__) and (E) tissue oxygen extraction (*Ṁ*_O_2__/*Q̇*). The vertical lines indicate when fish were injected with atropine or CPT at 10°C, and fish were injected with CPT again at 14 and 18°C. Values are means±s.e.m. Light symbols show the data points for individual fish. In A, numbers indicate how many fish were remaining if less than started the trial. Note that data with *N*≤3 are not plotted.

Heart rate decreased from ∼75–80 beats min^−1^ at 100% air sat. to ∼42–48 beats min^−1^ at 40% air sat., whereas *V*_s_ increased (by ∼60–95%) in the three groups. However, this pattern was different from that observed for *Q̇* and *Ṁ*_O_2_ _(Fig. 3 and Table 2). Both of these parameters increased as water O_2_ levels were lowered, but then returned towards initial values (Fig. 3 and Table 2); changes that were associated with a transient increase in activity as the water's O_2_ level was gradually decreased (personal observations) as in Leeuwis et al. (2019). In Fig. 3, it appears that *Ṁ*_O_2__/*Q̇* increased greatly between 100 and 90% air sat. in the CPT group. However, this was due to a large increase in *Ṁ*_O_2__/*Q̇* in two fish, and thus, the differences in *Ṁ*_O_2__/*Q̇* between 100% and 40% air sat. before drug injection were not significant (Table 2).

Atropine injection increased *f*_H_ considerably at 40% air sat. (by ∼55%; from ∼42 to 69 beats min^−1^). However, this only resulted in an increase in *Q̇* of 25% because *V*_s_ fell by approximately 28%, and there was no change in *Ṁ*_O_2_ _as the increase in *Q̇* was balanced by a decrease in *Ṁ*_O_2__/*Q̇* (Fig. 3). In contrast, no significant effects of CPT injection were observed at 10°C and 40% air sat. (Fig. 3, Table 2). As the atropine-injected fish were warmed, *f*_H_ increased by ∼20 beats min^−1^ (to ∼98 beats min^−1^ at 18°C) and this value was approximately twice that observed in the other groups at this temperature (Fig. 3, Table 2). However, *Q̇* fell continuously in these fish because of an almost linear decrease in *V*_s_ as temperature was increased; the *V*_s_ in this group much lower than in the control fish and in those treated with CPT at higher temperatures (Fig. 3). Despite the higher values of *Q̇* after atropine injection, and the decrease in *Q̇* in these fish as temperature rose, *Ṁ*_O_2__ was not different in this group when compared with the control group. This was because although *Ṁ*_O_2__/*Q̇* was initially considerably lower post-injection than in control fish, it began to increase at approx. 12°C concurrent with the temperature-dependent fall in *Q̇* [i.e., it appeared that changes in *Q̇* resulted in reciprocal adjustments in *Ṁ*_O_2__/*Q̇* (Fig. 3)]. In contrast, *Ṁ*_O_2__/*Q̇* was not affected by the injection of CPT at 10°C, 14 or 18°C (Table 2), and as in the control group, did not change as temperature was increased.

This experiment was stopped at 20°C because of ethical concerns and because the determination of CT_max_ was not one of the goals of this study (i.e. as mandated by MUN's animal care committee). However, it was clear that allowing hypoxic salmon to increase *f*_H_ when warmed lowered their tolerance to high temperatures. Only four atropine-injected fish were left at 18°C, three at 19°C and only one fish made it to 20°C. In contrast, in the other two groups, all but one fish made it to 18°C and 10 out of 18 fish made it to 20°C.

## DISCUSSION

In these studies, we examined: (1) the cardiorespiratory responses of Atlantic salmon to moderate and severe hypoxia in combination with high temperatures; (2) the capacity of this system to meet this species' metabolic demands under these conditions; and (3) how they relate to upper thermal tolerance. In addition, we investigated what physiological mechanisms prevented this species from raising its *f*_H_ when exposed to severe hypoxia, and the implications for thermal tolerance. These studies provide several novel insights into how Atlantic salmon (fish) are impacted by these combined climate change-related stressors, including that cholinergically-mediated hypoxic bradycardia is key to preserving cardiac function at low water oxygen levels when high temperatures are simultaneously encountered, and that increased *Q̇* may not result in increased *Ṁ*_O_2__ because of limitations in tissue oxygen extraction under these combined challenges. The latter finding highlights that we need to better understand the interplay between cardiac function and tissue oxygen extraction in determining a fish's tolerance to various biotic and abiotic stressors.

### Effects of hypoxia on salmon cardiorespiratory function and thermal tolerance

There were no significant changes in cardiac function when salmon were exposed to 75% air saturated water at their acclimation temperature (12°C) compared with when measured under normoxic conditions. In contrast, exposure to water with an air saturation of 50 (experiment 1) and 40% (experiment 2) decreased *f*_H_ by approx. 23 and 30 beats min^−1^, respectively, compared with *f*_H_ at 100% air sat. (Tables 1 and 2; Figs 1A and 3A). These decreases in *f*_H_ are somewhat greater than those reported in previous studies conducted on Atlantic salmon and other salmonids at similar temperatures and/or levels of hypoxia (Gamperl et al., 1994; Holeton, and Randall, 1967; Morgenroth et al., 2021; Sandrelli and Gamperl, 2023), but not unexpected because hypoxia tolerance varies between salmonid populations (Faust et al., 2004). Nonetheless, our results are in agreement with the literature in that *Q̇* was maintained or increased slightly when the salmon became bradycardic due to a compensatory increase in *V*_s_, and that *Ṁ*_O_2__ was not affected. This increase in *V*_s_ is likely due to a number of factors, including an increase in the time available for cardiac filling, higher values for central venous pressure in fish exposed to hypoxia and improved myocardial contractility at lower heart rates (i.e. the negative force–frequency affect on fish cardiac muscle; Sandblom and Axelsson, 2005; Shiels et al., 2002). Furthermore, the maintenance of *Ṁ*_O_2__ is consistent with previous work showing that the Atlantic salmon's critical oxygen tension (*P*_crit_) at the temperatures used in this study (10–12°C) is approximately 30–35% air sat. (Hvas and Oppedal, 2019; Remen et al., 2013, 2016).

As the salmon were warmed, increases in *f*_H_, *Q̇* and *Ṁ*_O_2__/*Q̇* (i.e. scope and maximum values) were all lower (although not significantly) in fish exposed to 75 versus 100% air sat. and AS was reduced by 45% in the former (Table 1, Fig. 1). However, the salmon's CT_max_ was not significantly less (23.6±0.9 vs 26.1±0.7°C in normoxic fish). In contrast: (1) fish exposed to warming at 50% air sat. (experiment 1) had a CT_max_ value (21.3±0.7°C) that was 4.8°C less than in normoxic fish, and this lower thermal tolerance was associated with scope for *f*_H_, *Q̇* and AS values that were 57, 35 and 60% lower than those measured in normoxic fish (Table 1); and (2) salmon challenged with rising temperatures at 40% sat. (in experiment 2) had a CT_max_ estimated at only ∼18°C based on the number of fish left at each temperature (see Results), and that values for cardiorespiratory parameters changed very little with further increases in temperature (Table 2, Fig. 3).

There is only one other study that provides comprehensive data on how the combined effects of these two environmental challenges influence fish cardiorespiratory function and thermal tolerance (i.e. Leeuwis et al., 2021). Thus, it is difficult to interpret how the changes observed in this study on Atlantic salmon compare with other fish species. Nonetheless, that *f*_H_ and *Q̇* did not increase in severely hypoxic 10°C-acclimated Atlantic salmon is consistent with what was observed in sablefish (*Anoplopoma fimbria*) acclimated to 10–11°C and exposed to the same level of hypoxia (Leeuwis et al., 2021). Collectively, these data suggest that severely hypoxic fish have little to no capacity to increase *Q̇* when exposed to increasing temperatures. However, the sablefish was still able to increase *Ṁ*_O_2__ by 70% as temperature was increased to their CT_max_ owing to a large increase in *Ṁ*_O_2__/*Q̇* (2.4-fold) that was not significantly different from that in fish tested under normoxia (Leeuwis et al., 2021). Thus, it appears that hypoxia tolerant species may regulate tissue oxygen extraction differently under oxygen limiting conditions than hypoxia intolerant species (i.e. salmon) and that this physiological response plays a major role in determining the sablefish's tolerance to warm temperatures [i.e., CT_max_ was only reduced by approximately 3°C (from ∼25 to 22°C) in this species when tested at 40% air sat.]. What mechanism(s) allow for the large enhancement in O_2_ extraction in the sablefish (and possibly other hypoxia-tolerant fishes) is/are not known. However, it has been suggested that plasma-accessible carbonic anhydrase may enhance root effect-mediated O_2_ offloading from haemoglobin in this species (Harter et al., 2019; Leeuwis et al., 2019).

With regard to the effects of hypoxia on the upper thermal tolerance of fishes, our results provide several insights. First, Ern et al. (2016) showed that the CT_max_ of lumpfish (*Cyclopterus lumpus*) and red drum (*Sciaenops ocellatus*) warmed at the same rate as in the present study (2°C h^−1^) was independent of oxygen availability over a broad range of water oxygen levels (100% to ∼30% and 20% air sat., respectively) despite large reductions in AS. Our results are largely in agreement with their findings, as AS was only significantly related to CT_max_ in fish exposed to 50% air sat. in experiment 1 (Fig. 2D) and support the growing body of evidence that physiological functions other than those governing oxygen supply capacity primarily determine the CT_max_ of fishes (Andreassen et al., 2022; Ern et al., 2023; Haverinen and Vornanen, 2020; Vornanen, 2020). Furthermore, they support the use of the oxygen limit for thermal tolerance [PCT_max_; which is the water oxygen tension (*P*w_O_2__) where an organism's CT_max_ starts to decline; Ern et al., 2016] to assess the oxygen sensitivity of upper thermal limits in water-breathing ectotherms and the susceptibility of their upper thermal limits to environmental hypoxia.

Second, the data of Ern et al. (2016) suggest that CT_max_ does not decrease until very close to a fish's *P*_crit_, and that at this *P*w_O_2_,_ the decrease in a fish's CT_max_ is ≤2°C. However, the CT_max_ of the Atlantic salmon was already 4.8°C lower than in normoxic fish at 50% air sat. and based on the *x*-intercept of the AS–CT_max_ relationship for these fish, it is estimated that the Atlantic salmon's CT_max_ at *P*_crit_ (where AS is 0 by definition) was ∼18.5°C in this study (see Fig. 2D). This latter value is ∼7.5°C lower than in normoxic fish. These limited data suggest that the upper thermal tolerance of hypoxia-intolerant fish may be more dependent on AS and that PCT_max_ is higher in such fish. This hypothesis needs to be tested experimentally, but such studies could help clarify under what conditions constrained oxygen supply capacity (i.e. as characterized in the ‘oxygen and capacity limited thermal tolerance’ concept; Pörtner and Knust, 2007; Pörtner, 2010; Pörtner et al., 2017) is relevant to upper thermal tolerance (i.e. when thermal tolerance is oxygen dependent versus oxygen independent) and to what extent upper thermal tolerance will be impacted by conditions of reduced oxygen availability that are routinely encountered by wild fishes.

### Heart rate control during severe hypoxia and implications for thermal tolerance

In experiment 2, we used cholinergic and A_1_-adenosinergic receptor antagonists to examine the role of these two modulators of cardiac function in hypoxia-mediated bradycardia, and in preventing/limiting *f*_H_ increases with rising temperature in the Atlantic salmon. Injection of atropine into severely hypoxic salmon at 10°C resulted in a large (26 beat min^−1^; ∼60%) increase in *f*_H_ (Table 2, Fig. 3A). However, *f*_H_ was still approx. 10 beats min^−1^ less than that observed in normoxic (saline injected) fish. This suggests that some other mechanism(s) was/were partly responsible for the lower *f*_H_ measured in severely hypoxic salmon. While, it is clear that adenosine (the stimulation of A_1_-adenosinergic receptors) was not involved as CPT did not affect the fish's *f*_H_ at 10°C (Table 2, Fig. 3; see below), it is not known at present why *f*_H_ was lower in these fish. Although Altimiras et al. (1997) suggest that non-adrenergic/non-cholinergic (NANC) control of heart function is likely to be more important when fish face environmental challenges, Fritsche and Nilsson (1990) report that *f*_H_ is slightly higher, not lower, in cod (*Gadus morhua*) exposed to acute hypoxia after both atropine and sotalol (a β-adrenergic receptor blocker) administration. Furthermore, although Faust et al. (2004) show that the *f*_H_ of isolated (*in situ*) rainbow trout hearts falls when exposed to severe hypoxia, no such effect was observed in this same species by Overgaard et al. (2004) or in the Atlantic cod (Petersen and Gamperl, 2010a). These two latter studies, and Schwieterman et al. (2023) suggest that hypoxia does not have direct negative chronotropic effects on *f*_H_.

In mammals, adenosine stimulates cardiac A_1_ receptors, and this leads to a decrease in *f*_H_ owing to hyperpolarization of sinus nodal cells as well as cells of the atrioventricular (AV) node. It has also been shown that this purine diminishes the effect of sympathetic nervous activation (and subsequent β_1_-stimulation), and thus, has an ‘anti-adrenergic’ effect by lowering intracellular cAMP levels (see Koeppen et al., 2009). Based on these findings, and because the administration of adenosine and A_1_-receptor agonists decrease *f*_H_ both *in vivo* and *in vitro* in fishes (Aho and Vornanen, 2002; Sundin and Nilsson, 1996; Sundin et al., 1999; Vornanen and Tuomennoro, 1999), it seemed likely that the hypoxic bradycardia observed in Atlantic salmon at 12°C was at least partially mediated by the stimulation of A_1_ receptors. However, this was not the case. Administration of the A_1_ receptor specific blocker CPT did not result in tachycardia at any of the test temperatures (10, 14 and 18°C; Fig. 3A), and there are several possible explanations for the lack of this antagonist's effect on the salmon's *f*_H_. MacCormack and Driedzic (2004, 2007) and MacCormack et al. (2006) reported that adenosine does not accumulate in the hearts of severely hypoxic sculpin (*Myoxocephalus scorpius*) or armoured catfish (*Liposarcus pardalis*) during severe acute hypoxia, and Vornanen and Tuomennoro (1999) showed that the cardiac effects of adenosine are weak in the crucian carp (*Carassius carassius*) at physiological concentrations. In addition, research on mammals has shown that the effects of adenosine on *in vivo f*_H_ can be masked by changes in cholinergic tone in response to sites of action other than the heart (Belloni et al., 1989). However, it is unlikely that the latter phenomenon explains the lack of an effect of CPT in this study, as adenosine receptor blockade after atropine administration failed to influence *f*_H_ in sculpin (MacCormack and Driedzic, 2004). Thus, it is likely that myocardial adenosine levels during acute severe hypoxia do not get high enough in the salmon heart to influence cardiac function. Nonetheless, these data do not preclude the possibility that adenosine has significant chronotropic effects on the heart of some fish under oxygen limiting conditions. For example, Stecyk et al. (2007) reported that administration of the adenosine antagonist aminophylline resulted in a modest tachycardia in common carp exposed to anoxia for 12.5 h. It is possible that oxygen deprivation for this extended period resulted in sufficient adenosine accumulation to influence *f*_H_, and that this enabled this species to sustain cardiac function through a better balance between ATP production and demand.

Bradycardia due to increased cholinergic tone on the heart occurs in most fishes (i.e. excluding hagfishes and some lungfishes) in response to acute severe hypoxia (Gamperl and Driedzic, 2009; Iversen et al., 2010; MacCormack and Driedzic, 2007; Wood and Shelton, 1980). Research has shown that the benefits of this decrease in *f*_H_ are not related to branchial gas exchange (Perry and Deforges, 2006) but to supporting and/or improving myocardial oxygenation and performance in the face of reduced blood oxygen levels (Farrell, 2007). However, increases in *f*_H_ are key to a fish's capacity to meet energetic demands as temperatures increase (Farrell et al., 2009; Eliason and Anttila, 2017), and thus, it is possible that if hypoxia-induced bradycardia prevented *f*_H_ from rising with temperature, a fish's upper thermal tolerance (CT_max_) could be negatively impacted. Indeed, Leeuwis et al. (2021) reported that hypoxia-induced bradycardia dominated over the requirement for increasing *f*_H_ during warming of the sablefish and suggested that this ‘physiological chain of command’ limits the fish's cardiac response to the latter stressor, and ultimately, its upper thermal tolerance. Clearly, the present research shows that this is not true for the Atlantic salmon, as allowing *f*_H_ to increase greatly during severe hypoxia resulted in a decrease in this species' thermal tolerance.

In atropine-treated salmon, *V*_s_ fell immediately after injection of this pharmacological agent and continued to decrease as *f*_H_ increased further with temperature (Fig. 2C). The initial post-injection decrease in *V*_s_ was expected given the strong inverse relationship between *V*_s_ and *f*_H_ in fishes (Farrell and Smith, 2017; Farrell et al., 1996; Shiels et al., 2002). However, the steady decrease in *V*_s_ (and *Q̇*) was not anticipated as these parameters were unchanged as temperature increased in control fish, even though *f*_H_ also rose slightly (Fig. 3). In addition, Ekström et al. (2014) showed that *V*_s_ was maintained in atropine-treated normoxic trout at intermediate temperatures and increased as temperature approached the trout's CT_max_. The post-injection *f*_H_ of atropine-treated fish (∼70 beats min^−1^) and the low venous blood O_2_ levels concomitant with severe hypoxia in salmonids (Holeton and Randall, 1967), suggest that the salmon heart was already struggling to maintain myocardial oxygenation and cardiac function and that additional increases in *f*_H_ magnified this effect.

A particularly novel finding was that adjustments in *Q̇* in atropine-injected hypoxic salmon were associated with reciprocal changes in *Ṁ*_O_2__/*Q̇*. Specifically, this latter parameter fell sharply immediately after atropine injection, but increased as *V*_s_ and *Q̇* declined with temperature (Fig. 3). These data strongly suggest that there was a diffusion limitation at the tissues of these fish that prevented increases in *Ṁ*_O_2__ with flow (*Q̇*). The relationship between oxygen extraction efficiency and blood flow has been studied and modelled for over a century in mammalian systems/tissues and shows that reductions in capillary transit time heterogeneity (CTTH) are key to counteracting the drop in the fraction of oxygen extracted that invariably occurs during hyperemia; specifically, increasing the number of capillaries that are perfused (i.e. decreasing CTTH) prevents deceases in capillary transit time (CTT) from negatively impacting tissue oxygen extraction and ultimately limiting oxygen consumption (Jespersen and Østergaard, 2012; Kalliokoski et al., 2004; Østergaard, 2020). In experiment 2, the salmon were exposed to a severe level of hypoxia during which *Q̇* was maintained at normoxic levels and most gut blood flow (which constitutes 30–40% of *Q̇* under normoxic conditions in most fishes) would have already been redirected to critical tissues before the fish reached 40% air sat. (Seth et al., 2011; Axelsson and Fritsche, 1991). Furthermore, the capillaries in these tissues were likely to be maximally or near maximally dilated given the large reduction in trout arterial oxygen partial pressure and content at this level of hypoxia (Claireaux et al., 2008; Gamperl et al., 1994; Holeton and Randall, 1967). If under this scenario one assumes that CTTH could not be increased further (i.e. all capillaries were maximally or near maximally dilated), transit time would decrease considerably if *Q̇* was increased (i.e. after atropine injection; see Fig. 3B) and it is possible/probable that this hyperemia-induced reduction in CTT would lead to a large enough decrease in the fraction of oxygen extracted (OEF) to result in no change, or even a decrease, in oxygen delivery to the tissues (i.e. oxygen extraction that becomes inefficient toward short mean transit times). Importantly, the above discussion raises the possibility that the cholinergically mediated bradycardia not only protects cardiac function under conditions of oxygen limitation as detailed by Farrell (2007), but also ensures that cardiac workload and oxygen demand do not increase beyond values that would benefit tissue (animal) oxygen consumption.

### Summary and perspectives

A number of studies have examined how previous exposure of organisms to hypoxia or high temperatures influences their tolerance to the other condition (i.e. whether organisms possess ‘cross-tolerance’), and the plastic physiological responses that might permit species' resilience to climate change (for a review, see Earnheart et al., 2022). However, there have been few studies that have examined the capacity of fishes to tolerate simultaneous exposure to these two important climate change stressors (e.g. see Del Rio et al., 2019; Ern et al., 2026; Leeuwis et al., 2021; Lima et al., 2024; Schwieterman et al., 2019), which are becoming more common in the marine environment (Keeling et al., 2010). In this paper, we show that acute thermal tolerance (CT_max_) of the Atlantic salmon (an ecologically and economically important marine fish species) is quite sensitive to water oxygen levels and may be reduced by more than 7°C as they approach their *P*_crit_. We also provide important and novel insights into the physiological processes and mechanisms that determine the thermal tolerance of fish when oxygen levels become limiting.

As first shown by Ern et al. (2016) for red drum and lumpfish, the Atlantic salmon's AS and CT_max_ were not significantly related until water oxygen levels were reduced substantially. Thus, our results are in agreement with a number of previous studies which suggest that the acute upper thermal tolerance limits of fishes are not always linked to the capacity for oxygen delivery (i.e. aerobic scope) and that physiological functions other than those governing oxygen supply capacity primarily determine a fish's CT_max_ (e.g. see Ern et al., 2016, 2023; Jutfelt et al., 2018; Wang et al., 2014). However, comparison of our data with Ern et al. (2016) reveals that the *P*w_O_2__ at which thermal tolerance becomes oxygen dependent (i.e. PTC_max_) shows significant interspecific variation. The physiological basis/es for this are not known. However, identifying them and determining the PCT_max_ of important species or taxa could significantly improve our capacity to predict the impacts of climate change on fish populations and lead to more effective conservation and management strategies (Lefevre et al., 2021; McKenzie et al., 2016).

Our results also reveal that cholinergic tone (but not adenosinergic modulation) prevents salmon from increasing *f*_H_ once hypoxia-induced bradycardia has been initiated. However, they do not support the assumption of Leeuwis et al. (2021) that the inability to increase *f*_H_ at low oxygen levels limits a fish's ability to tolerate higher temperatures. In fact, an increase in *f*_H_ under conditions of severe hypoxia had the opposite effect (i.e. it decreased thermal tolerance), and this was due to both the negative effects of elevated *f*_H_ on the pumping capacity of the heart (*V*_s_) and of *Q̇* on tissue oxygen extraction. The former effect supports evidence from several studies suggesting that there are a number of benefits to heart function of lowering *f*_H_ when oxygen is limited (see Farrell, 2007). Furthermore, it suggests that while cholinergic tone on the heart has no or limited (a decrease in the temperature of cardiac arrhythmias and of CT_max_ of ≤2°C) benefit for a fish's upper thermal tolerance under normoxic conditions (Ekström et al., 2014, 2019, 2021; Gilbert et al., 2019), this mechanism of slowing *f*_H_ may be critical for survival when fish face increased metabolic demands under conditions of limited oxygen supply. In support of this hypothesis, it has been reported that while sea bass (Dupont-Prinet et al., 2009) and Atlantic cod (Petersen and Gamperl, 2010a) increase *f*_H_ greatly when swum to their critical swimming speed under normoxic conditions, fish exposed to 45–50% air sat. show no or minimal increases in *f*_H_ when exercised. It is possible (likely) that increased cholinergic tone prevents/limits the normal exercise-induced increase in *f*_H_, and that this allows the heart to continue pumping and for increases in *Q̇* with swimming/activity. However, whether this occurs, and if so, the specific benefit(s) of this constraint on *f*_H_ during exercise need to be investigated.

With regard to the inverse relationship between tissue oxygen extraction and *f*_H_ in severely hypoxic salmon, this is a particularly novel finding which suggests that the potential benefits of increased *Q̇* (hyperemia) to tissue oxygen uptake are not realized under these conditions because of a concomitant reduction in the OEF. In this paper, we speculate that this is because transit time is limiting at the low arterial blood oxygen levels concomitant with severe hypoxia. However, we have a poor understanding of the scope for changes in *Ṁ*_O_2__/*Q̇* in fishes, what determines inter-specific differences in this parameter, and what mechanisms regulate it during acute and chronic exposure to changing environmental conditions. For example, although it has been suggested that the importance of *Ṁ*_O_2__/*Q̇* in supporting a species' increased metabolic demands may be related to its maximum *f*_H_ (or scope for *f*_H_) or hypoxia tolerance (Leeuwis et al., 2021), there appears to be no clear pattern when changes in cardiorespiratory parameters during a CT_max_ trial are compared between the American eel (*Anguilla anguilla*; Claësson et al., 2016), Nile tilapia (E.S.P. and A.K.G., unpublished results), sablefish (Leeuwis et al., 2021), Atlantic cod (Gollock et al., 2006), steelhead trout (Motyka et al., 2017) and Atlantic salmon (present study). This is despite increases in *Ṁ*_O_2__/*Q̇* that are greater than *Q̇* in some of these species, a 2-fold difference in maximum *f*_H_ (∼60–125 bpm) between species, and a wide range in hypoxia tolerance (i.e. the sablefish and tilapia are extremely hypoxia tolerant whereas the salmonids and the cod are considered to be hypoxia intolerant/sensitive). One of the reasons for this may be that tissue oxygen extraction in these studies was not determined by measuring differences in the O_2_ content of the arterial and venous blood but using the Fick equation. This method of calculating *Ṁ*_O_2__/*Q̇* does not take into account cutaneous gas exchange, which can vary greatly between fish species (Kirsch and Nonnotte, 1977; Nonnotte and Kirsch, 1978) and leads to an underestimation of a fish's venous oxygen reserve to deal with changes in oxygen demand (Claësson et al., 2016; Farrell, 1984). Additional studies are clearly needed, where heart function, *Ṁ*_O_2__, *C*a_O_2__ and *C*v_O_2__ are measured in a number of species under varying abiotic and biotic challenges, before we can better understand the degree to which tissue oxygen extraction contributes to *Ṁ*_O_2_ _in fishes and its functional significance and evolutionary underpinnings.

## Supplementary Material

10.1242/jexbio.249594_sup1 Supplementary information
