# Supplementary material for: It's a good thing that severely hypoxic salmon (Salmo salar) have a limited capacity to increase heart rate when warmed
Source: J Exp Biol. 2025 Mar 6;228(5):JEB249594. doi: 10.1242/jeb.249594 (PMC11925397; doi:10.1242/jeb.249594)
Supplement: Supplementary information [file jexbio-228-249594-s1.pdf]

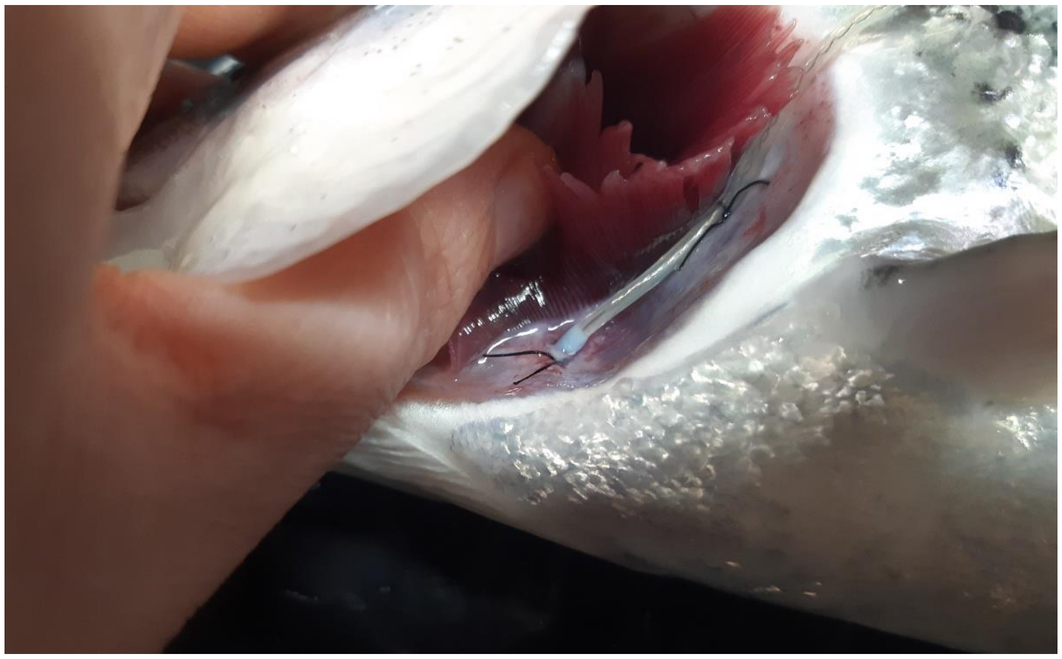

Fig. S1. Picture of the inside of the salmon's opercular cavity showing the location of the Doppler flow probe, and the first stitch to secure the flow probe lead to the fish.

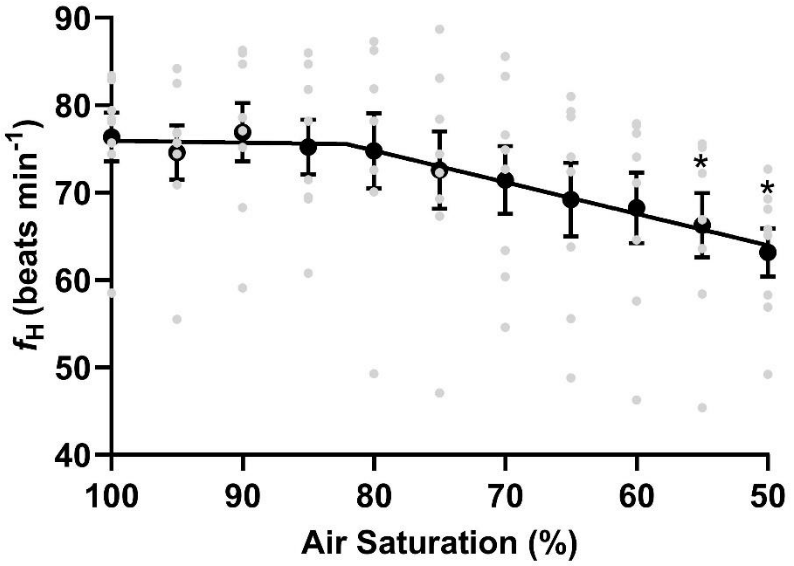

Fig. S2. Heart rate (f_H) in eight Atlantic salmon at 12°C as water in their respirometer was decreased to 50% air saturation. An asterisk indicates when f_H was significantly different from the value at 100% air saturation. The line was fitted on the individual data using a segmental ('broken stick') regression. Values are means $\pm$ 1 standard error of the mean (s.e.m.). Light symbols show the data points for individual fish

Journal of Experimental Biology • Supplementary information

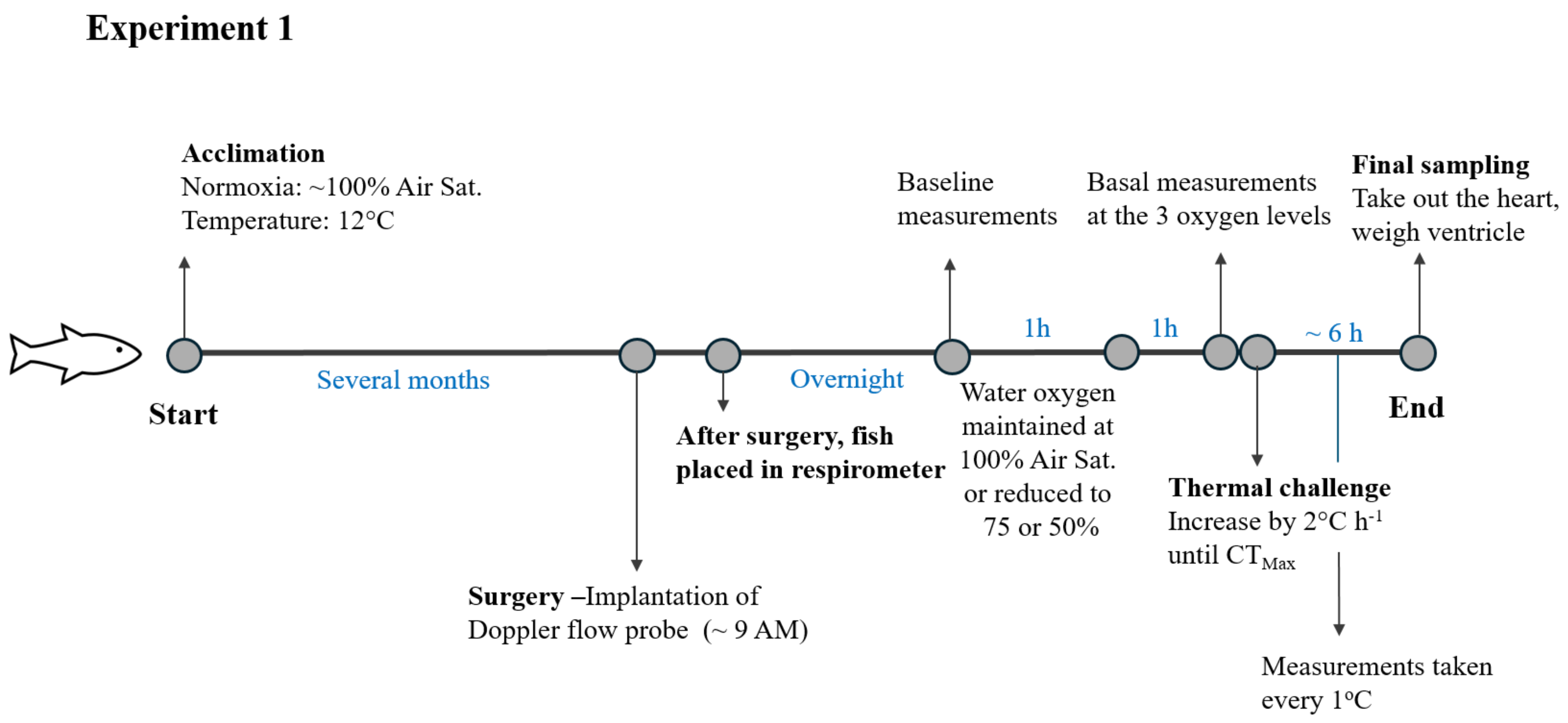

Fig. S3. Schematic diagram showing the sequence of events used to examine the effects of various levels of water oxygenation on cardiac function and thermal tolerance in the Atlantic salmon.

Journal of Experimental Biology • Supplementary information

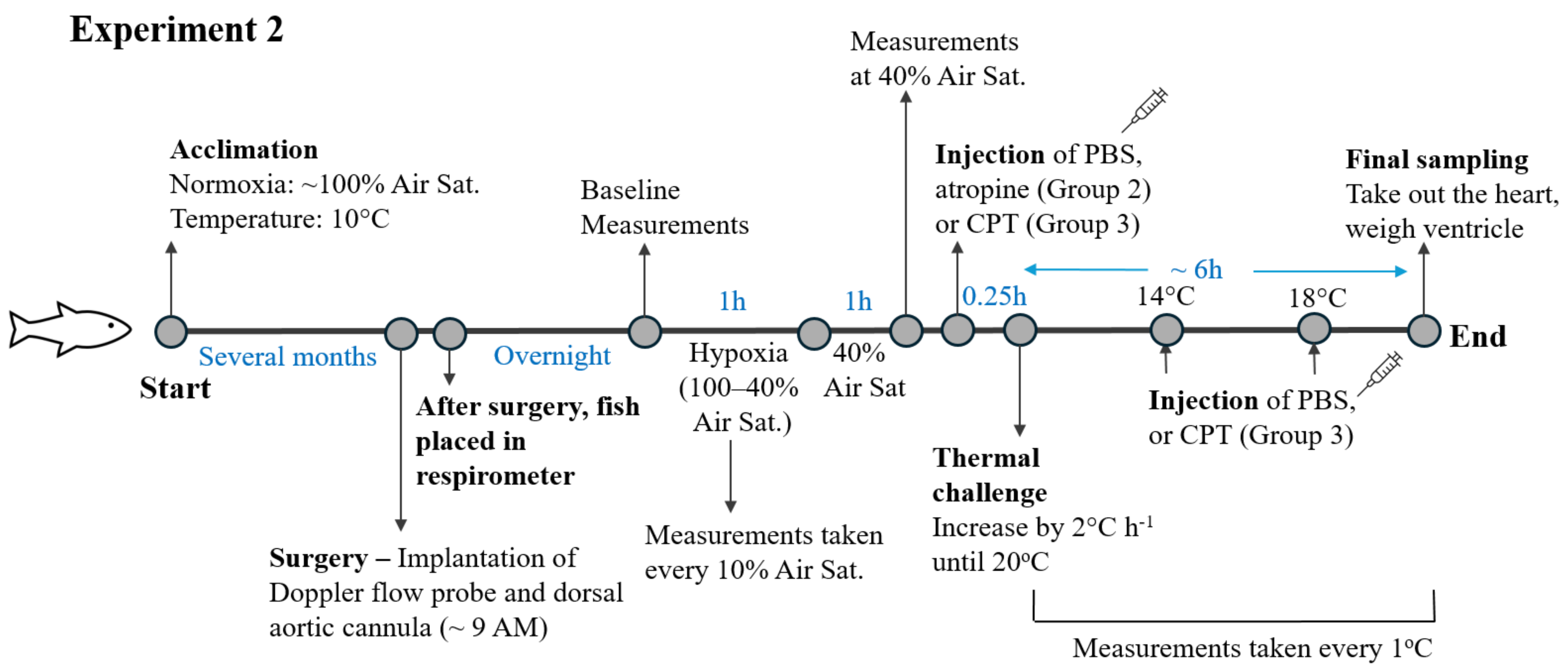

Fig. S4. Schematic diagram showing the sequence of events used to examine the role of cholinergic nervous tone and adenosinergic control on the heart in determining the salmon's cardiorespiratory function and thermal tolerance when exposed to the combined stressors of hypoxia and an acute increase in temperature. PBS = phosphate buffered saline; CPT = 8-cyclopentyltheophylline.

Journal of Experimental Biology • Supplementary information

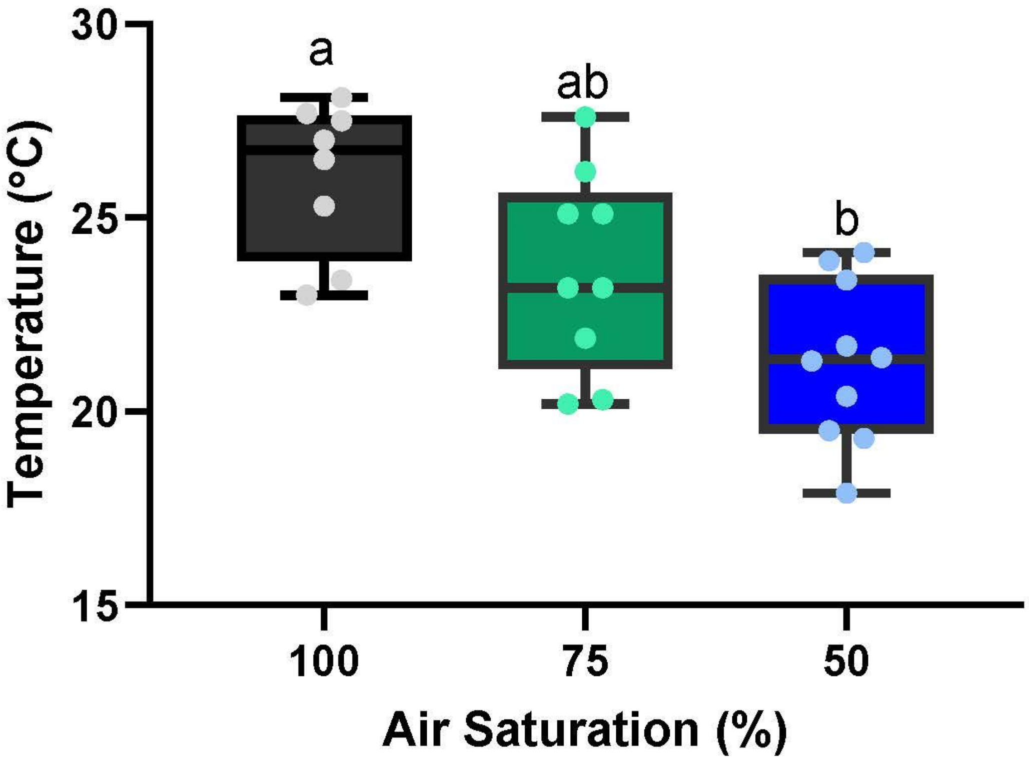

Fig. S5. Critical thermal maximum (CT_{Max}) values for Atlantic salmon (N = 9) during normoxic conditions (100% air saturation), and when fish were held at 75 and 50% air saturation, respectively. In the CT_{Max} test, fish were warmed at 2°C h^{-1}. Data are means $\pm$ 1 standard error of the mean (s.e.m.). N = 9. Dissimilar lower-case letters indicate significant differences ($P < 0.05$) as determined using a one-may ANOVA. Light symbols show the data points for individual fish.

Journal of Experimental Biology • Supplementary information
